# Feasibility and accuracy of real-time three-dimensional echocardiography in evaluating the aortic valve in children

**DOI:** 10.1186/s43044-019-0037-8

**Published:** 2020-01-07

**Authors:** Dina Adel Ezzeldin, Alaa Mahmoud Roushdy, Abdallah Ahmed Abdallah, Azza Abdallah El Fiky

**Affiliations:** Cardiology Department, Faculty of Medicine, Ain Shams University Hospital, Cairo, Egypt

## Abstract

**Background:**

Aortic valve assessment by 2D transthoracic echocardiography is a relatively complex task owing to the unique anatomical features of the left ventricular outflow tract and its dynamic nature. We aimed to evaluate the accuracy of 3D transthoracic echocardiography [3D TTE] in assessing the aortic valve in children.

**Results:**

The first group included 11 males and six females, with a mean age of 5.76 ± 6.39 years. All of these patients had aortic valve disease with a bicuspid variant. The second group included seven males and seven females, with a mean age of 4.4 ± 4.05 years. All of these patients had normal aortic valve morphology and had another congenital cardiac anomaly. The aortic valve annulus was assessed using the three modalities; 2D, 3D echocardiography in the vertical and horizontal diameters, and angiography. The aortic valve area was measured by 2D and 3D echocardiography using multiplane reformatted mode. The results of the analysis were then compared. They revealed that 3D echocardiographic measurement of the aortic annulus (horizontal diameter) correlated better with angiography than 2D and 3D (vertical diameter) echocardiographic measurements. There was a significant difference between the aortic valve area measured by 2D echocardiography and that measured by 3D echocardiography among the two groups, 2D echocardiography seems to underestimate the true aortic valve area.

**Conclusion:**

The study concluded that 3D TTE with multiplane reformatted mode allows a more accurate assessment of the aortic valve when compared to 2D echocardiography and this correlates better with the angiographic findings.

## Background

The most common cause for congenital valvular aortic stenosis is bicuspid aortic valve, with an estimated prevalence of 1–2% in the general population [1].

Previous studies performed in adults with severe aortic stenosis suggested an underestimation of the aortic annulus diameter with two-dimensional (2D) echocardiography compared with tomography [2].

Only few studies have addressed this subject in the pediatric population. Real-time three-dimensional echocardiography (3D-TTE) is an emergent non-invasive technique, useful in the evaluation of cardiac chamber volumes, mass, and left ventricular wall motion as well as in the analysis of morphology and function of heart valves [3].

3D TTE has the potential to become a new noninvasive modality that complements conventional techniques in the assessment of aortic stenosis. It can be used for assessing the aortic valve and root morphology and also to calculate the valve area [4, 5].

From the 3D pyramidal data set, a cropping plane aligned exactly parallel to the flow-limiting aortic orifice, viewed in long axis, can be used to obtain a short-axis 3D image of the aortic valve orifice. The aortic valve orifice area then can be measured accurately by direct planimetry or by using the software system provided by the manufacture. Another advantage of 3D echocardiography is the ability to estimate the aortic valve orifice area in patients who have combined aortic stenosis (AS) and other stenotic lesions such as tandem like supra valvular stenosis and discrete sub aortic stenosis [6, 7].

Calculation of the aortic valve area by continuity equation with conventional echocardiography is based on several assumptions: left ventricular outflow tract geometry is circular and adequately described by its antero-posterior diameter in 2D parasternal long-axis view; the 2D cut plane is parallel to left ventricular outflow tract spatial orientation; Doppler tracings of left ventricular outflow tract flow are acquired at the same site where the left ventricular outflow tract diameter is measured.

These assumptions have proven to be inaccurate, especially when the heart is rotated or horizontal. Since the left ventricular outflow tract area is calculated based on the left ventricular outflow tract-squared diameter, the left ventricular outflow tract diameter becomes the greatest potential source of error in the continuity equation [8].

Moreover, both 3DE and multi-slice cardiac tomography (MSCT) studies showed that left ventricular outflow tract geometry assumes more frequently an elliptical configuration rather than a circular one [9]. Accurate left ventricular outflow tract size and geometry is not only critical for the quantitation of AS severity but also for correct annulus sizing during trans catheter aortic valve replacement, calculating stroke volume, and shunt ratio.

Direct planimetry of aortic annular/left ventricular outflow tract areas by 3D transesophageal echocardiography 3D TEE showed the best agreement with multi-slice cardiac tomography as gold standard, whereas the calculations based on the left ventricular outflow tract diameter measured by 2D transesophageal echocardiography or 3D transesophageal echocardiography led to significant area underestimation (by 16.4 and 12.9%, respectively) [9].

This translates into a reclassification of 10% patients from severe to moderate AS when using the calculated 3D transesophageal echocardiography [3D TEE] area and of 25% patients when using the planimetered 3D transesophageal echocardiography area [10].

In another study, the geometry of aortic complex (annular and left ventricular outflow tract areas, distances between aortic annulus and coronary ostia) quantified by 3D transesophageal echocardiography correlated very well with the measurements obtained by multi-detector computer tomography [11].

In addition, the left ventricular outflow tract area measured from biplane imaging by 3D TTE improved the accuracy of stroke-volume assessment and of AS severity assessment, when compared with invasive or direct planimetry [12, 13].

## Methods

The study included 31 patients. All patients were subjected to the following.

### Conventional 2D echocardiography

Standard 2D TTE was performed using Phillips iE33 echocardiography machine.

#### Sedation

In agitated infants and children, sedation with chloral hydrate aqueous solution (50 mg per kilogram) 15 min prior to the study was done.

#### Probe selection

A phased array S8-3 pediatric probe with frequency range from 8 to 3 MHz was used for infants below 1 year and a phased array S5-1 probe with frequency range from 5 to 1 MHz for infants older than one year and children.

#### Sequential analysis

Sequential analysis was applied for all patients to determine the situs, atrioventricular and ventriculoarterial connections, great vessel relation and abnormalities, ventricular dimensions and functions, state of cardiac valves, venous connections, and any intra cardiac shunts. Left ventricular hypertrophy was used as a useful marker for AS stenosis severity as other obstructive lesions were excluded.

Continuous wave Doppler was used to measure the velocity of flow across the stenotic aortic valve and was used to estimate the pressure drop across the valve using the simplified Bernoulli equation which states that *P* = 4V^2^, (where *P* is the peak instantaneous pressure gradient, in millimeters of mercury, across the obstructed aortic valve, and *V* is the peak flow velocity, in meters per second, distal to the obstructive orifice).

#### The views

**Fig. 1:**
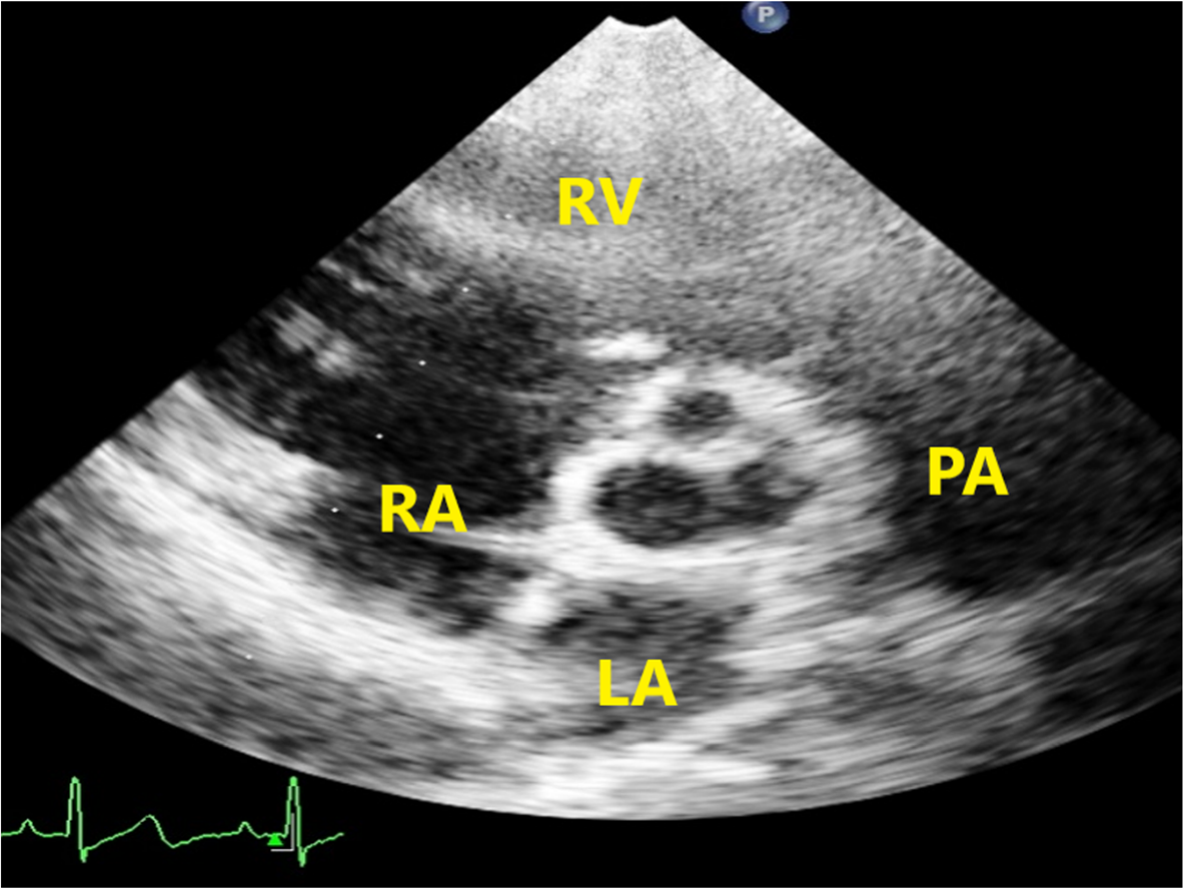
Parasternal short axis view showing the morphology of stenotic aortic valve in patient no. 8

The morphology of the stenotic aortic valve was evaluated from the parasternal short axis (Fig. 1) and the aortic annulus diameter was measured between the hinge points of the valve leaflets in the two-dimensional parasternal long axis view. The annulus was carefully and thoroughly scanned by transducer angulation so that the maximum possible diameter was obtained.

**Fig. 2:**
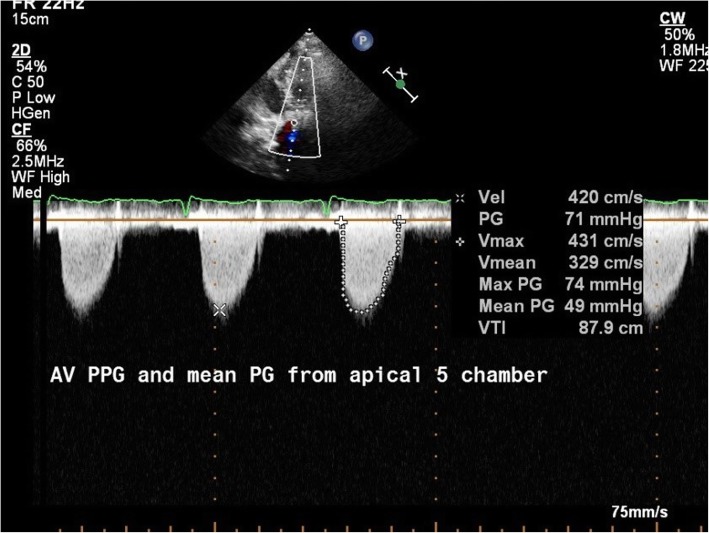
Continuous wave Doppler use in measurement of MPG and PPG across the aortic valve in patient no

**Fig. 3:**
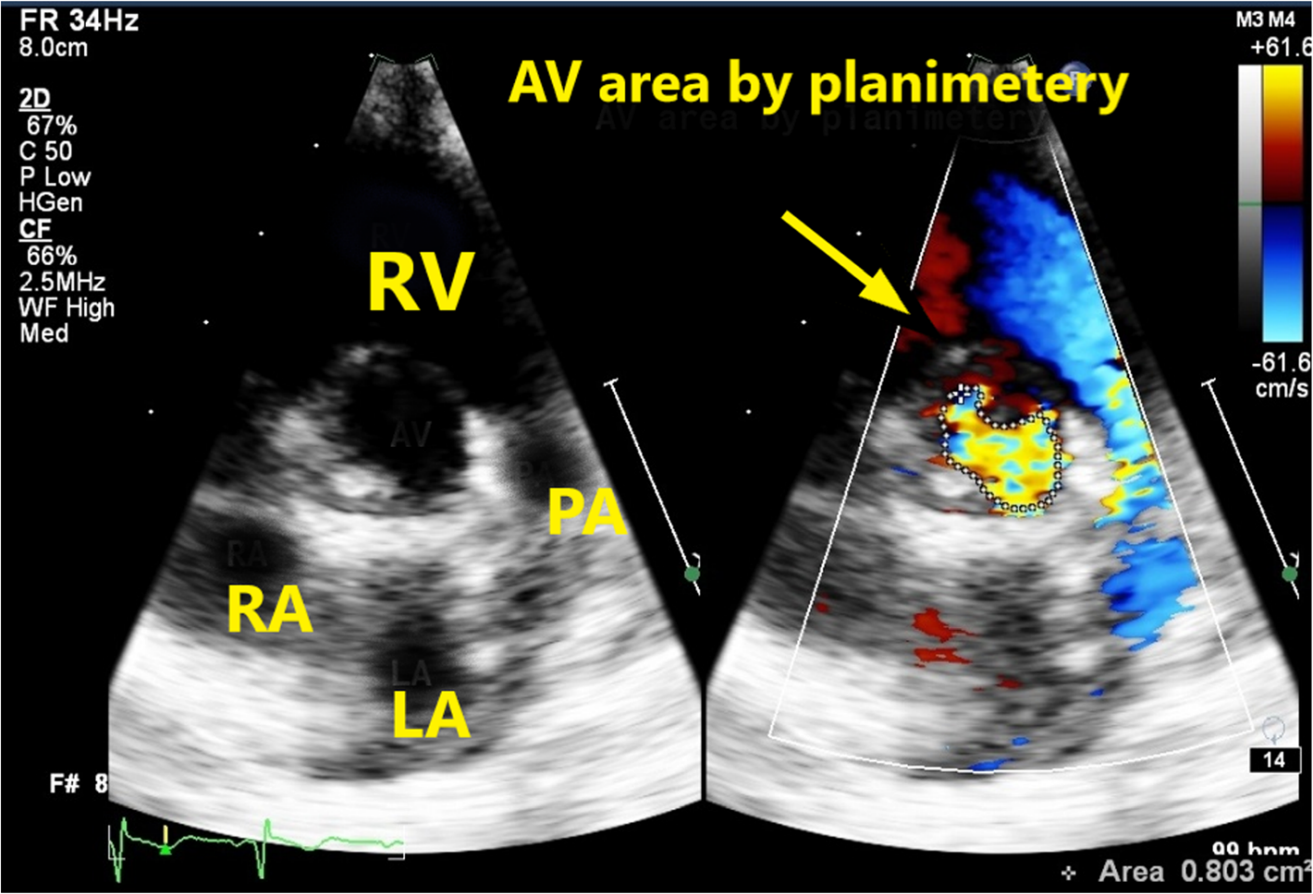
AVA was measured by direct planimetery in parasternal short axis view in patient no. 9

The peak pressure gradient and mean pressure gradient were obtained from different views (right parasternal, apical five chamber, and subcostal left ventricular outflow tract coronal views) with the cursor placed distal to the aortic valve and the maximum detected gradients were recorded (Fig. 2). Then the aortic valve area was measured by direct planimetery from the parasternal short axis view (Fig. 3) [14, 15].

### 3D echocardiography: [3D TTE]

After completing the 2D echocardiographic study, all the cases were subjected to 3D TTE echocardiography using the same Phillips iE33 echocardiography machine.

#### Probe selection

Full 3D study was done using a pediatric matrix probe X7-2 in infants and children up to 3 years of age with a frequency range from 7 to 2 MHz and an adult matrix probe X3-1 in children older than 3 years with frequency range from 3 to 1 MHz.

#### Modes of acquisition

All studies were ECG gated and two modes of acquisition were used and the aortic valve was assessed from the Para sternal long axis and para sternal short axis views:

1. Narrow angle live 3D imaging was first performed in all conventional 2D planes.
2. Full volume with stitched four cardiac cycles.

#### Method of analysis

**Fig. 4:**
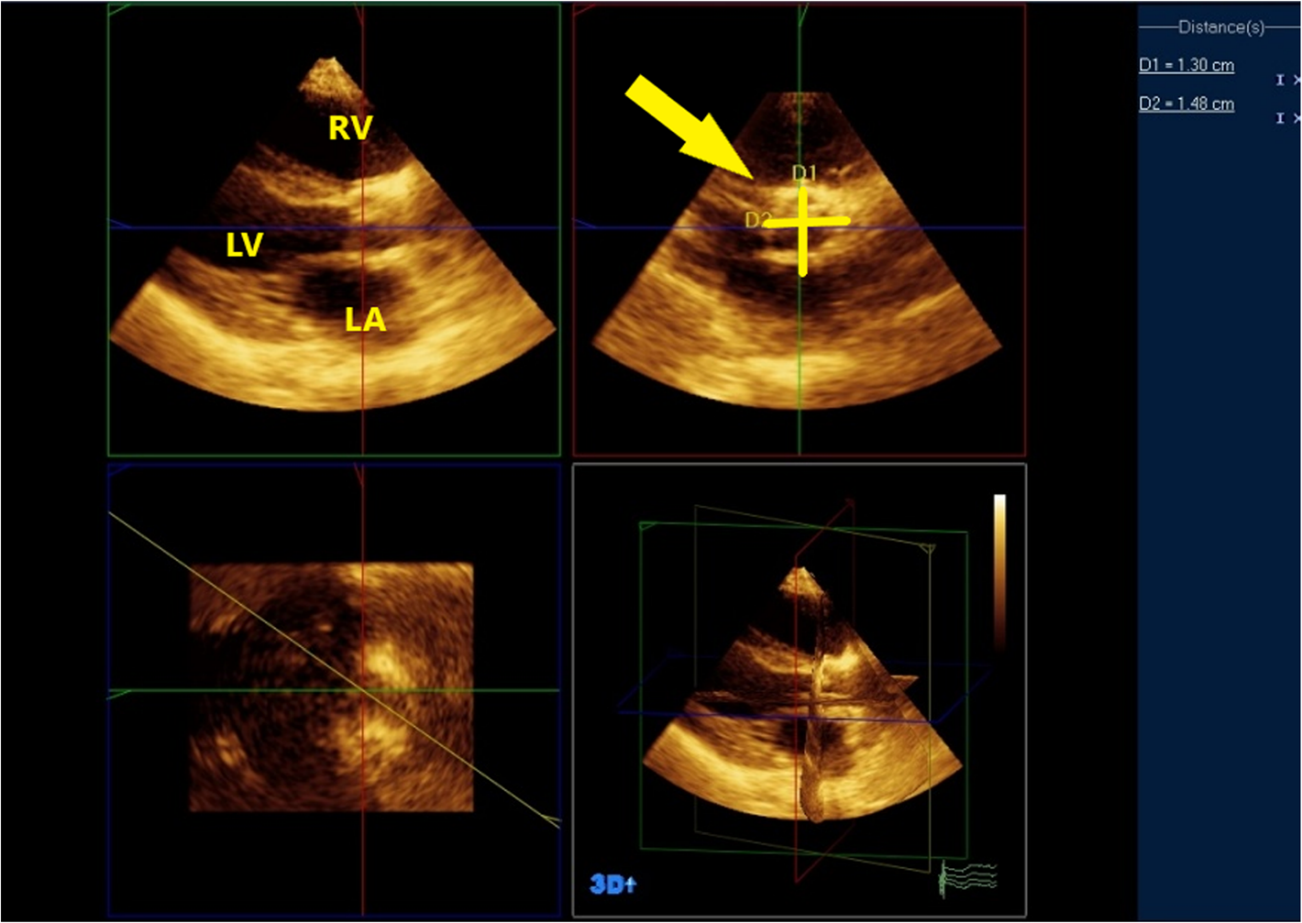
Aortic annulus was measured by 3D echocardiography in three orthogonal planes using MPR mode in patient no. 1

**Fig. 5:**
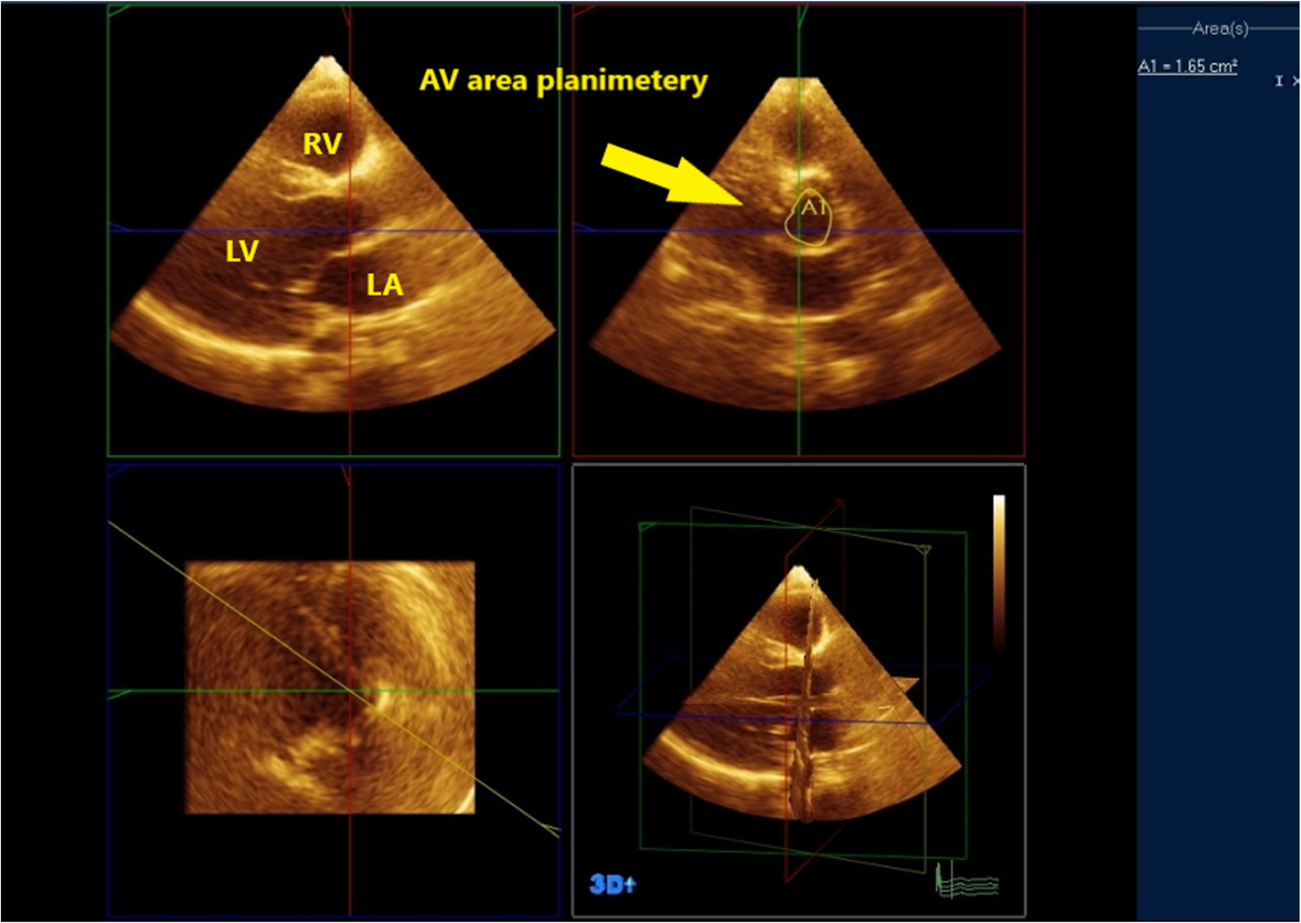
AVA was measured by direct planimetery in parasternal short axis view using MPR mode. In patient no. 1

Analysis was done off line using the Q-lab software and quantification system mostly on the echocardiography machine and rarely on the workstation using multiplane reformatted (MPR) mode with three independent orthogonal cutting planes. Two orthogonal planes were in the long axis of the left ventricle; the third plane was perpendicular to the two others and was moved at the insertion of aortic cusps to obtain a short-axis 2D plane of the aortic annulus. Care was taken to place the plane exactly at the insertion of the leaflets, as recommended. Horizontal and vertical diameters of the aortic annulus were measured (Fig. 4) then aortic valve area was measured by direct planimetery in parasternal short axis view (Fig. 5) [8–10].

### Catheterization data

The aortic valve annulus was measured after aortic root injection in left anterior oblique 40, and cranial angulation was gradually added, the cranial/caudal angulation was increased or decreased to avoid overlap with the spine or the descending aorta.

Using standard methodology, the measurement tool was calibrated to the respective diagnostic catheter for the aortic root measurements. Using the calibrated measurement tool, the annulus was measured at the lowest hinge point of the aortic valve leaflet [11].

### Statistical analysis

All data were gathered, statistically analyzed, and tabulated. All numerical variables were expressed as mean ± standard deviation (SD), and categorical variables were expressed as percentage (%). Changes in continuous variables were evaluated with the paired *t* test. For all analysis, a *P* value of < 0.05 was considered statistically significant. Spearman’s correlation coefficients were estimated to assess the correlation between 3D and catheter measurement. The Bland-Altman method was used to further investigate the differences between 2D and 3D measurements versus angiographic measurements.

## Results

This was a prospective observational study that included 31 patients who were referred for elective cardiac catheterization or follow up echocardiography in the period from November 2017 to May 2018.

### Patient characteristics

The study group was subdivided into two main groups; first one included 17 patients with diseased aortic valve and the other one included 14 patients with normal aortic valve morphology; the two groups were age and sex matched.

The first group included 11 males (64.7 %) and six females (35.3%), with a mean age of 5.76 ± 6.39 years (range 1 month to 27 years). All of these patients had aortic valve disease with a bicuspid variant.

The second group included seven males (50%) and seven females (50%), with a mean age of 4.4 ± 4.05 years (range 1–16 years). All of these patients had normal aortic valve morphology and had another congenital cardiac anomaly.

Regarding group 1 (diseased aortic valve), the weight of the patients ranged from 4 to 60 kg with a mean of 20.15 ± 13.12, the height of the patients ranged from 36 to 152 cm with a mean 93.53 ± 33.14, and the body surface area of the patients ranged from 0.26 to 1.59 m^2^ with a mean of 0.71 ± 0.33.

Regarding group 2 (normal aortic valve), the weight of the patients ranged from 8 to 55 kg with a mean of 18.21 ± 11.73, the height of the patients ranged from 70 to 162 cm with a mean 95.71 ± 25.85, and the body surface area of the patients ranged from 0.69 ± 0.3 m^2^ with a mean of 0.44–1.57. There was no significant difference between the two groups regarding age, sex, height, weight, and body surface area (BSA).

Patients with diseased aortic valve included eight patients with severe aortic stenosis who were referred for balloon aortic valvuloplasty and the annulus was measured angiographically (group 1a) and nine patients where the angiographic data were not present at the time of 3D echocardiographic evaluation (group 1b) as six of these patients underwent balloon aortic valvuloplasty before and came for follow up, and three patients were scheduled for balloon aortic valvuloplasty later on.

### Dimensional echocardiography data

All patients in the two groups had normal left ventricular internal dimensions for age with normal ejection fraction.

In group 1, the left ventricular end diastolic dimension ranged from 32.64 ± 9.13 mm with a mean of 32 ± 8.58 mm, the left ventricular end systolic dimension ranged from 8 to 26 mm with a mean of 18.88 ± 5.88 mm, the interventriclar septal thickness ranged from 3.7 to 9 mm with a mean of 6.06 ± 1.62 mm, the posterior wall ranged from 3.7 to 9 mm with a mean of 6.06 ± 1.62 mm, the ejection fraction ranged from 62 to 80% with a mean of 72.41 ± 5.52%, the peak pressure gradient ranged from 20 to 105 mmHg with a mean of 59.41 ± 29.91 mmHg, the mean pressure gradient ranged from 11 to 68 mmHg with a mean of 31.82 ± 17.28 mmHg, the annulus ranged from 8 to 21 mm with a mean of 15.63 ± 4.56 mm, and the aortic valve area ranged from 0.3 to 1.9 cm^2^ with a mean of 1.1 ± 0.53.

In group 2, the left ventricular end diastolic dimension ranged from 18 to 43 mm with a mean of 32.71 ± 7.75 mm, the left ventricular end systolic dimension ranged from 11 to 28 mm with a mean of 20.36 ± 5.53 mm, the IVS ranged from 3 to 8 mm with a mean of 6.06 ± 1.62 mm, the posterior wall ranged from 3 to 7 mm with a mean of 4.96 ± 1.15 mm, the ejection fraction ranged from 58 to 77% with a mean of 66.71 ± 6.41, and the peak pressure gradient ranged from 8 to 19 mmHg with a mean of 14 mmHg. ±

The aortic valve annulus ranged from 9 to 21 mm with a mean of 15.79 ± 3.85 mm, and the aortic valve area ranged from 1.7 ± 0.47 with a mean of 0.8–2.5 (Table 1).

**Table 1:** 2D echocardiographic data

		Group 1 Diseased AV	Group 2 Normal AV	Independent *t* test	
				*t*/X^2^	*P* value
LVESD	Mean ± SD	18.88 ± 5.88	20.36 ± 5.53	− 0.714	0.481
	Range	8–26	11–28		
LVEDD	Mean ± SD	32.64 ± 9.13	32.71 ± 7.75	− 0.026	0.980
	Range	18–49	18–43		
EF	Mean ± SD	72.41 ± 5.52	66.71 ± 6.41	2.658	0.013
	Range	62–80	58–77		
IVSd	Mean ± SD	6.06 ± 1.62	5.18 ± 1.41	1.594	0.122
	Range	3.7–9	3–8		
Pw	Mean ± SD	6.06 ± 1.62	4.96 ± 1.15	2.120	0.043
	Range	3.7–9	3–7		
PPG	Mean ± SD	59.41 ± 29.91	14 ± 3.76	5.628	0.000
	Range	20–105	8–19		
MPG	Mean ± SD	31.82 ± 17.28	8.79 ± 2.29	4.938	0.000
	Range	11–68	5–13		
Ann 2D	Mean ± SD	15.63 ± 4.56	15.79 ± 3.85	− 0.102	0.920
	Range	8–21	9–21		
AVA 2D	Mean ± SD	1.1 ± 0.53 mm.	1.7 ± 0.47	− 3.264	0.003
	Range	0.3–1.9 mm	0.8–2.5		

### Three-dimensional echocardiography data

Regarding the Q lab feasibility, all cases included in the study were feasibly assessed by Q lab and the following measurements were obtained:

Regarding group 1: the aortic annulus horizontal diameter had a mean of 18.32 ± 5.15 with a range of 11–27 mm. The aortic annulus vertical diameter ranged from 8 to 22 mm with a mean of 15.56 ± 5.1 mm and the aortic valve area had a mean 1.24 ± 0.61 cm^2^ with a range of 0.3–2.1 cm^2^.

Regarding group 2: the aortic annulus horizontal diameter had a mean of 17.79 ± 4.35 with a range of 10–25 mm. The aortic annulus vertical diameter ranged from 9 to 22 mm with a mean of 16.04 ± 3.9 mm and the aortic valve area had a mean 1.83 ± 0.54 cm^2^ with a range of 0.8–3 cm^2^ (Table 2).

**Table 2:** Comparison of aortic valve 3D echocardiographic data between the two groups

		Group 1 Diseased AV	Group 2 Normal AV	Independent *t* test	
		No. = 17	No. = 14	*T*	*P* value
Ann 3D H	Mean ± SD	18.32 ± 5.15	17.79 ± 4.35	0.310	0.759
	Range	11–27	10–25		
Ann 3D V	Mean ± SD	15.56 ± 5.1	16.04 ± 3.9	− 0.287	0.776
	Range	8–22	9–22		
AVA 3D	Mean ± SD	1.24 ± 0.61	1.83 ± 0.54	− 2.807	0.009
	Range	0.3–2.1	0.8–3		

### Angiographic data

Regarding first group the diameter of the aortic annulus for eight patients was measured in the left anterior oblique cranial view and ranged from 10 to 24 mm with a mean of 17.24 ± 5.54 mm, while in the second group it was measured in all patients and ranged from 10 to 26.5 mm with a mean of 17.57 ± 4.31.

### Comparison between annulus by 2D and 3D in diseased AV group

A paired *T* test was used to compare the aortic annulus measured by 2D, 3D echocardiography horizontal and vertical diameters. As it was almost impossible to determine whether the longer or the shorter dimension measured by 3D echocardiography is the dimension that corresponds to that measured by 2D echocardiography, the comparison was made with both dimensions separately.

There was no significant difference between the aortic annulus measured by 2D echocardiography and that measured by 3D echocardiography vertical diameter (*P* = 0.885)*.* There was a significant difference between the horizontal diameter measured by 3D echocardiography and that measured by 2D echocardiography (*P* < 0.001)*.* Also there was a significant difference between the horizontal and the vertical diameters measured by 3D echocardiography (*P* < 0.001) (Table 3).

**Table 3:** Comparison between aortic annulus measured by 2D and 3D echocardiogram in group 1

	Group 1 Diseased AV group			P1*	P2**	P3***
	Ann 2D	Ann 3D H	Ann 3D V			
Mean ± SD	15.63 ± 4.56	18.32 ± 5.15	15.56 ± 5.10	0.000	0.885	0.000
Range	8–21	11–27	8–22			

*P1: Ann 2D versus Ann 3DH done by paired *t* test, **P2: Ann 2D versus Ann 3DV done by paired *t* test, **P3: Ann 3DH versus Ann 3DV done by paired *t* test

### Comparison between annulus by 2D, 3D, and angiography in group 1

There was a significant difference between the aortic annulus measured by 2D echocardiography and that measured by angiography (*P* = 0.000). Also there was a significant difference between the vertical diameter by 3D echocardiography and the diameter measured by angiography (*P* = 0.014)*.* There was a less significant difference between the horizontal diameter measured by 3D echocardiography and the annulus diameter measured by angiography (*P* = 0.043) (Table 4).

**Table 4:** Comparison between aortic annulus measured by 2D and 3D echocardiogram versus annulus measured by angiography in group 1

	Group 1 Diseased AV group				P1*	P2**	P3***
	Ann 2D	Ann 3D H	Ann 3D V	Ann Angio			
Mean ± SD	15.63 ± 4.56	18.32 ± 5.15	15.56 ± 5.10	17.24 ± 5.54	0.000	0.043	0.014
Range	8–21	11–27	8–22	10–24			

*P1: Ann angio versus Ann 2D done by paired *t* test, **P2: Ann angio versus Ann 3DH done by paired *t* test, ***P3: Ann angio versus Ann 3DV done by paired *t* test

Nevertheless, there was a significant and strong linear correlation between 2D and 3D horizontal and vertical diameters versus angiography in the first group where *r* = 0.986 (*P* < 0.0001), *r* = 0.978 (*P* < 0.0001), and *r* = 0.955 (*P* < 0.0001) respectively.

### Comparison between annulus by 2D and 3D in the group 2

There was no significant difference between the aortic annulus measured by 2D echocardiography and that measured by 3D echocardiography vertical diameter (*P* = 0.468)*.* There was a significant difference between the horizontal dimension measured by 3D echocardiography and that measured by 2D echocardiography (*P* < 0.001). Also there was a significant difference between the horizontal and the vertical diameters measured by 3D echocardiography (*P* < 0.001).

### Comparison between annulus by 2D, 3D echocardiography versus angiography in group 2

There was a significant difference between the aortic annulus measured by 2D echocardiography and that measured by angiography (*P* = 0.000). Also there was a significant difference between the vertical 3D echocardiography and the diameter measured by angiography (*P* = 0.001)*.* Using the horizontal diameter measured by 3D echocardiography, there was no significant difference between it and the annulus diameter measured by angiography.

The correlation coefficients between 2D and 3D horizontal and vertical diameters versus angiography in the second group were *r* = 0. 986 (*P* < 0.0001), *r* = 0.978 (*P* < 0.0001), and *r* = 0.955 (*P* < 0.0001) respectively**.**

### Comparison between aortic valve area by 2D and 3D echocardiography in group 1

There was a significant difference between the aortic valve area measured by 2D echocardiography and that measured by 3D echocardiography (*P* = 0.000) (Table 5).

**Table 5:** Comparison between aortic valve area by 2D and 3D echocardiography in group 1

	Group 1 Diseased AV group		Paired *t* test	
	AVA 2D	AVA 3D	*t*	*P* value
Mean ± SD	1.10 ± 0.53	1.24 ± 0.61	− 4.429	0.000
Range	0.3–1.9	0.3–2.1		

### Comparison between aortic valve area by 2D and 3D echocardiography in normal valve morphology group

There was a significant difference between the aortic valve area measured by 2D echocardiography and that measured by 3D echocardiography (*P* = 0.005) using the paired *T* test (Table 6).

**Table 6:** Comparison between AVA by 2D and 3D echocardiography in the second group

	Normal valve morphology		Paired t-test	
	AVA 2D	AVA 3D	*T*	*P* value
Mean ± SD	1.70 ± 0.47	1.83 ± 0.54	− 3.347	0.005
Range	0.8–2.5	0.8–3		

The Bland-Altman analysis revealed that the mean difference between 2D, 3D horizontal and vertical diameters and the measurement obtained by angiography were (− 1.9 ± 2.4, 0.6 ± 2.3, and − 1.7 ± 1.9 mm) respectively.

## Discussion

Echocardiography has evolved into the most predominant diagnostic imaging technique in valvular heart disease [8–11, 13]. Over the last five decades, the diagnostic capability of echocardiography has increased dramatically from M-mode to two-dimensional imaging. Recent advances in ultrasound instrumentation and computer technology have led to the evolution of three-dimensional echocardiography, introducing a new era in cardiovascular imaging [10].

Every imaging technique in cardiology aims at complete visualization and comprehensive assessment of cardiac morphology and pathology. Analysis of the heart in motion in all three or four (including time) dimensions can therefore further facilitate and enhance the diagnostic capabilities of echocardiography.

Three-dimensional echocardiography is still in its evolution and at the phase of early adaptation with respect to its clinical application. It should complement current echocardiographic techniques by providing better understanding of the topographical aspects of pathology and refined definition of the spatial relationships of intra-cardiac structures [10].

The present study was designed to evaluate the role of live 3D echocardiography in the assessment of aortic valve morphology compared to 2D echocardiography and angiography.

Goland et al. estimated the aortic valve area with good accuracy and reproducibility in patients with severe aortic stenosis, using 3D-guided and real-time 3D echocardiography [11].

A recent study in a population of adults with aortic stenosis showed that 3D transesophageal echocardiography was an accurate method for evaluating aortic root geometry and should be considered as a non-invasive no radiation alternative to multi detector computed tomography [13].

Real-time three-dimensional echocardiography has also been reported to give accurate information about valvular dimensions and morphology in congenital bicuspid aortic valve, with an excellent correlation with surgical assessment [16]. These findings suggest that this technique can be used routinely in the assessment of the aortic valve in children.

The first study to assess measurement of the aortic valve effective orifice area by three-dimensional echocardiography was made by Tara Bharucha et al. in 2011 on 12 children (mean age 4.4–5.0 years). The study assumed that measurement of effective orifice area by three-dimensional echocardiography (3DE) may by more reliable than continuity equation or two dimensional echo [17].

The study assessed measurement of aortic valve effective orifice area by three-dimensional echocardiography in children with AS before and after balloon aortic valvuloplasty and compared results with change in aortic valve gradient. Using three-dimensional echocardiography multiplanar review mode, valve annulus diameter, area, and effective orifice area were measured and compared, with change in aortic gradient and degree of aortic insufficiency [18].

The study concluded that three-dimensional echocardiography facilitates effective orifice area measurement in pediatric AS and correlates with change in aortic valve gradient after balloon valvuloplasty [19]. In the current study, we measured aortic valve area by 2D and 3D planimetery in two groups with and without diseased aortic valve and found that 3D measurements were larger than 2D measurements which is most probably due to the noncircular nature of the orifice which can be appreciated by three-dimensional echocardiography as well as the thickening of the leaflets.

Further complicating 2D assessment of the effective aortic orifice area is that the plane of the stenotic orifice may be “slanted,” i.e., out of the plane that is orthogonal to the long axis of the aortic root. These problems can be solved with three-dimensional echocardiography, offering better visualization of the left ventricular outflow tract; three-dimensional echocardiography may increase accuracy of effective orifice area planimetry [20, 21].

In 2012, David Black et al. reviewed the use of three-dimensional echocardiography with multiplanar reformatting in children with congenital aortic stenosis undergoing percutaneous balloon aortic valvuloplasty to assess its accuracy in measuring the aortic valve annulus and any influence it may have on balloon sizing [22, 23].

They concluded that three-dimensional echocardiography with multiplane reformatted mode allows a more accurate assessment of the aortic valve annulus compared to two-dimensional echocardiography, which may reduce the tendency to undersize balloon choice [24] and these results are in agreement with our results which stated that angiographic measurement of the annulus correlates better with 3D horizontal diameter than 3D vertical diameter and 2D measurements; this is due to the fact that two-dimensional echocardiography measurements assess the valve in only one plane in contrast to three-dimensional echocardiography with multiplane reformatted mode which allows the assessment of the aortic valve annulus in a number of orthogonal planes [25].

In the current study, there was no significant difference between two-dimensional echocardiography measurement and three-dimensional vertical diameter. This could be explained by the fact that they both capture the aortic valve orifice in the same plane. We found that three-dimensional horizontal aortic valve annulus diameter differs significantly than 2D and 3D vertical diameters; this can be explained by the fact that the annulus is not circular but oval in shape and that 3D could measure it from different planes. Comparing 3D echocardiography measured aortic valve horizontal annulus diameter to aortic annulus measured by angiography yielded similar measurements with a statistically insignificant difference; this can be attributed to the angle at which we measured the annulus in the left anterior oblique projection.

Thomas Cogneta et al. in 2013 assessed the feasibility of three-dimensional transthoracic echocardiographic planimetry in congenital bicuspid aortic valve in children and evaluated the influence of valvular asymmetry and aortic valve area on stenosis severity. Seventy consecutive children with bicuspid aortic valve were included in this prospective single center study. Using the multiplanar review mode, surfaces were measured by planimetry. The degree of stenosis was assessed by instantaneous aortic Doppler. The authors concluded that three-dimensional echocardiographic planimetry is a feasible and reproducible method for assessing aortic surfaces in congenital bicuspid aortic valve.

When compared with catheterization and two-dimensional echocardiography (2DE), three-dimensional echocardiography was proven to be accurate and reproducible for the quantification of aortic valve area [26, 27].

Moreover, the multiplane reformatted mode derived from 3D echocardiography has been reported to be more precise than two-dimensional echocardiography because it overcomes the physiological deformation of aortic annulus during the cardiac cycle [28].

In our study, we measured the aortic valve area by 2D and 3D echocardiography then measured the aortic annulus by two-dimensional and three-dimensional echocardiography in two orthogonal planes (horizontal and vertical) and compared them with each other and with the gold standard angiography. We found that aortic valve area by three-dimensional echocardiography tended to be larger than that measured by two-dimensional echocardiography and this can be explained that the smallest opening, its shape (round or slit like), and precise orientation (anterior–posterior orientation or medial–lateral orientation) can be better assessed by three-dimensional echocardiography.

We compared the aortic annulus measurement by 2D, 3D echocardiography with angiography in both patients with diseased aortic valve as well as normal aortic valve morphology. Romain Martin. et al. in 2013 compared two- and three-dimensional transthoracic echocardiography for measurement of aortic annulus diameter in a normal pediatric population. Thirty children without heart disease were prospectively included. The long axis and short axis annulus diameters of 3D echocardiography were significantly different in both systole and diastole in agreement with our study which found significant difference between horizontal and vertical diameter of the annulus; they found also that 2D aortic annulus diameter in systole was significantly smaller than the maximum obtained in 3D which is similar to our study and that is related to the fact that the shape of aortic annulus as previously mentioned is more oval than circular and is also asymmetrical even in healthy valves [29–31].

In 2014, Rolf Alexander Jánosi et al. reported quantitative analysis of aortic valve stenosis and aortic root dimensions by 3D echocardiography in patients scheduled for trans-cutaneous aortic valve implantation. They performed two-dimensional transthoracic echocardiography (2D-TTE), real-time 2D transesophageal echocardiography (RT2D-TEE), and real-time 3D transesophageal echocardiography in 71 consecutive patients referred for transcatheter aortic valve replacement. Real-time 3D transesophageal echocardiography multiplanar reconstruction was used to measure aortic root parameters, including left ventricular outflow tract (LVOT) diameter and area, aortic annulus diameter, aortic annulus area, and aortic valve area. Real-time 3D transesophageal echocardiography methods for planimetry and the left ventricular outflow tract-derived continuity equation for the estimation of aortic valve area showed a good correlation [32].

The aortic annulus diameter was measured by 2D transthoracic echocardiography, 2D transesophageal echocardiography, and real-time 3D transesophageal echocardiography, and the annulus area was measured by 2D transesophageal echocardiography, and real-time 3D transesophageal echocardiography successfully in all patients. The aortic annulus area measured by 2D- transesophageal echocardiography was significantly smaller than that calculated by real-time 3D transesophageal echocardiography (3.63 ± 0.72 cm^2^ vs. 4.05 ± 0.71 cm^2^; *P* < 0.05). There was a high correlation between the planimetry estimated by 2D-transesophageal echocardiography and real-time 3D transesophageal echocardiography (*r* = 0.81). The real-time 3D transesophageal echocardiography data underlined again that the aortic annulus has an ellipsoid shape and is not circular [33].

Also in our study, the annulus diameter measured by 2D echocardiography and 3D echocardiographic vertical diameter was significantly smaller than 3D echocardiographic horizontal diameter in both pathological valve group (15.63 ± 4.56, 15.56 ± 5.10 vs. 18.32 ± 5.15) and normal valve group (15.79 ± 3.85, 16.04 ± 3.90 vs. 17.79 ± 4.35). We found that that the angiographic measurements were better correlated with 3D horizontal diameter than 3D vertical diameter and 2D measurements in both groups.

## Limitations

The main limitation of our study is the small sample size. Also the continuity equation does not form a part of our echocardiography laboratory’s standard protocol for assessment of aortic valve area and was not available for analysis in this study and for comparison with three-dimensional echocardiography planimetry.

Another limitation is that we did not correlate the used balloon size to the 2D, 3D echocardiographic or angiographic measurements and estimate the degree of aortic regurgitation after the procedure.

## Conclusion

The results showed that 2D echocardiography measurements were significantly smaller than 3D and angiographical measurements. This underlines the accuracy of 3D in the quantitative assessment of the aortic annulus. As a supplement to angiographical measurements, 3D echocardiography with multiplanar reconstruction may reduce the tendency to undersize balloon choice. Due to the asymmetry of the aortic annulus, real-time 3D echocardiography may better capture its geometry and has to be considered as an additional technique for a multimodal assessment of the aortic root. Adequate assessment of the aortic annulus diameter has other implications in surgery.

The Ross procedure requires preoperative sizing of the annulus and good visualization of the aortic morphology. Surgical aortic valve repair also requires the accurate measurement of aortic root dimensions to guide prosthesis selection, size, and design.

## Data Availability

The datasets used and/or analyzed during the current study available from the corresponding author on reasonable request.

## Abbreviations

2D: Two-dimensional echocardiography
3D: Three-dimensional echocardiography
Angio: Angiography
AR: Aortic regurgitation
AS: Aortic stenosis
AV: Aortic valve
AV: Atrioventricular
AVA: Aortic valve area
BSA: Body surface area
CH: Congestive heart failure
CHD: Congenital heart disease
EF: Ejection fraction
EOA: Effective orifice area
EST: Exercise stress test
FS: Fractional shortening
FV 3DE: Full volume three-dimensional echocardiography
HOCM: Hypertrophic cardiomyopathy
IVS: Interventricular septum
LAO: Left anterior oblique
LV: Left ventricle
LVEDD: Left ventricular end diastolic dimension
LVESD: Left ventricular end systolic dimension
MPR: Multiplane reformatted mode
MV: Mitral valve
PDA: Patent ductus arteriosus
PV: Pulmonary valve
RT3DE: Real-time three-dimensional echocardiography
RV: Right ventricle
RVOT: Right ventricular outflow tract
TEE: Transesophageal echocardiography
TTT: Treatment
TV: Tricuspid valve
VA: Ventriculoarterial
VCA: Vena contracta
VSD: Ventricular septal defect
